# Perception of Web-Based Didactic Activities During the COVID-19 Pandemic Among Anesthesia Residents: Pilot Questionnaire Study

**DOI:** 10.2196/31080

**Published:** 2022-03-31

**Authors:** Ala Nozari, Shivali Mukerji, Ling-Ling Lok, Qingrou Gu, Lauren Buhl, Sanjay Jain, Rafael Ortega

**Affiliations:** 1 Boston University School of Medicine Boston, MA United States; 2 Beth Israel Deaconess Medical Center Boston, MA United States

**Keywords:** resident education, COVID-19, barriers to education, didactic, medical education, online education, web-based education, virtual training, anesthesiology residents, medical residents, pandemic, virtual didactics

## Abstract

**Background:**

Physical and social distancing recommendations aimed at limiting exposure during the COVID-19 pandemic have forced residency programs to increasingly rely on videoconferencing and web-based resources.

**Objective:**

In this pilot study, we aimed to explore the effects of the COVID-19 pandemic on residency training experience, and to delineate the perceived barriers to the successful implementation of web-based medical education.

**Methods:**

A 19-item survey was compiled and distributed electronically using Qualtrics. This anonymous survey included information on the training level of each resident, their participation in formal didactics before and during the pandemic, and their perception of the ease and limitations of virtual didactics. The resident’s opinions on specific educational resources were assessed, and the effectiveness of new delivery methods on resident engagement and learning was examined.

**Results:**

Thirty anesthesiology residents were surveyed, 19 of whom agreed to participate in the pilot study. One participant with incomplete responses was excluded, yielding a final cohort of 18 respondents. Most residents (56%, 10/18) reported that the COVID-19 pandemic negatively affected their residency training. The time spent on didactic training and independent studies was, nevertheless, not affected by the pandemic for 90% (16/18) of respondents. Nonetheless, 72% (13/18) of residents were less engaged during virtual lectures in comparison to in-person didactics. Important limitations included distraction from the physical environment (67%, 12/18), internet instability (67%, 12/18), less obligation to participate (44%, 8/18), technical difficulty and unmuted microphones (33%, 6/18, each), and people speaking over each other (28%, 5/18). Despite these limitations, most residents stated that they would like to keep a combination of virtual didactics including live Zoom lectures (56%, 10/18), prerecorded web didactics (56%, 10/18), and virtual ground rounds via Zoom (50%, 9/18) as the “new normal.”

**Conclusions:**

Despite important limitations listed in this report, anesthesia residents would like to keep a combination of virtual lectures and presentations as the new normal after the COVID-19 pandemic.

## Introduction

Didactic activities are an important component of anesthesia residency training and are required by the Accreditation Council for Graduate Medical Education [1]. As frontline workers were involved in aerosol producing procedures with the highest risk of transmission during the peak of the COVID-19 pandemic in Boston, Massachusetts, anesthesia residents were heavily affected by the added stress of potential infection and changing clinical responsibilities, while adapting to a modified didactic curriculum and remote learning [2]. Physical and social distancing recommendations [3] aimed at limiting exposure during the COVID-19 pandemic led to the cancellation of face-to-face didactic activities [4], forcing residency programs to increasingly rely on videoconferencing and web-based resources. While limiting the risk of exposure during a pandemic, virtual meetings and web-based resources allow the residents to work closely with other trainees and faculty within their institution, in addition to breaking geographic restrictions through cost-effective collaborative networks for educators across the nation and beyond [5]. The learning experience may be limited by technical barriers, lack of engagement, distraction from physical environments, and other factors that have yet not been fully explored. Course development and delivery are also critical for success. In a study comparing the differences in virtual versus in-person training for occupational therapists, researchers found that while knowledge acquisition did not differ between the two groups, participant’s satisfaction rating was higher for the in-person group than for the web-based group owing to lack of in-person interactions, lack of ease in networking, and synchronous interactions in the web-based setting. The same study also found that participants appreciated the ease of accessibility and flexibility provided by the web-based learning modules [6]. Another study assessing the development of empathy in premedical students found a significant increase after a 2-week remote learning course. However, since comparisons to empathy development in a similar in-person course are missing, it is difficult to determine whether a similar or greater increase in empathy would not have been observed in an in-person setting [7]. The authors could not identify previous studies on the effect of distance learning on significant components of postgraduate medical education such as knowledge acquisition, engagement, and the development of empathy. It is hence unclear whether virtual meetings and didactics can satisfy the educational needs of residents and ensure the quality of experience that is required for their academic, clinical, and professional growth, and further research is needed to address this gap.

In this pilot study, we aimed to explore the effects of the COVID-19 pandemic on the residency training experience and delineate the perceived barriers to the successful implementation of web-based medical education within our anesthesiology residency program, using a self-reported, anonymous survey.

## Methods

### Ethical Considerations

The institutional review board at the Boston Medical Center determined that this study (H-40592) qualified for an exemption, under the policies and procedures of the Human Research Protection Program.

### Study Overview

Boston Medical Center is the largest safety net level 1 trauma center in eastern Massachusetts. Because of the significant impact of the pandemic and large number of patients with COVID-19, all elective surgeries were suspended and residents were deployed to airway and intubation teams, ventilator management teams, critical care and obstetric services, or emergency surgeries. All lectures, grand rounds, case conferences, and morbidity mortality discussions were transitioned to web-based class sessions using a cloud-based teleconferencing software platform (Zoom). On July 24, 2020, after the initial peak in COVID-19 cases at our hospital, we distributed a 19-item survey to all residents in our training program (N=30) using Qualtrics Survey Software [8]. Following the initial survey distribution, 2 weekly electronic reminders were sent to the residents. The survey was closed for responses on August 7, 2020. This anonymous survey included information on the training level of each resident, their participation in formal didactics before and during the pandemic, and their perception on the ease and limitations of virtual didactics. Activities under “formal didactics or independent learning” included lectures and topics on basic and advanced anesthesia such as clinical pharmacology, cardiovascular and respiratory physiology, perioperative medicine, and considerations for complex disease states and surgical procedures. Special topics related to the pandemic such as airway management in patients with suspected or confirmed COVID-19 were, of course, added to the curriculum, but the basics of perioperative medicine and critical care remained unchanged. In addition to these lectures, anesthesia residents also participated in regular Grand Rounds and Case Conferences, discussing topics of interest to the perioperative medicine and special considerations in patients with comorbidities and other perioperative concerns.

The survey was developed by a formative committee within our department using a modified Delphi technique with a three-generation telephone interview, personal interview, and conference [9]. After obtaining ethics approval form the institutional review board, the survey was piloted with 3 academic physicians (experts) and 3 trainees (respondents) in our department, and iteratively revised to improve clarity and construct validity. The context of the survey was determined by its main purpose of examining the effectiveness of virtual teaching to anesthesia residents, taking into consideration the environmental, educational, cultural, and social context of medical education. Close-ended questions were mainly chosen to better standardize, collect, and analyze the data and because of their high reliability, and some open-ended questions were also included to provide freedom of expression and allow for unanticipated responses.

All data are reported as frequencies and percentages. The time spent on formal didactics or independent learning before and during the COVID-19 pandemic were considered ordinal data and compared using the Friedman nonparametric test. Free-text comments were categorized into representative themes and analyzed using conventional qualitative content analysis [10].

## Results

We surveyed our anesthesiology residents (N=30), 70% of whom (range 21-30) responded and 63% (N=19) agreed to participate in this pilot study. One participant was excluded owing to incomplete responses, making the final cohort comprise 18 participants (60%). A larger proportion of junior residents (clinical anesthesia year 1 or CA-1) responded to the survey (8/18, 44%) compared to the senior residents (5/18, 28%, for CA-2; 5/18, 28%, for CA-3) (Table 1). The response rate was 100% for all questions except for the question of the impact of COVID-19 on residency training, which yielded a 67% response rate (12/18).

Most residents (56%, 10/18) reported that the COVID-19 pandemic negatively affected their residency training. Four residents (22%, 4/18) felt that their training was not affected, and another 22% (4/18) of respondents felt that it was positively affected by the pandemic. Hands-on training in the operating rooms was reported to be reduced for 56% (10/18) of responding residents, a majority of whom (70%, 7/10) reported a >50% reduction.

No significant differences were observed in the time spent on formal didactics (*P*=.50) or independent study (*P*=.70) before and during the COVID-19 pandemic, irrespective of the seniority of the residents. Additionally, a higher proportion of residents reported spending 0-5 hours on didatics during than before the COVID-19 pandemic (61%, 11/18 vs 50%, 9/18, respectively). This change reflects an equal decrease in residents who report spending 5-10 hours on formal didactics before than during the COVID-19 pandemic (44%, 8/18 vs 33%, 6/18, respectively).

**Table 1:** Distribution of respondents by year in residency training and time spent on didatics before and during the COVID-19 pandemic (N=18).

Time spent on didactics (hours)		Respondents before the COVID-19 pandemic, n (%)	Respondents during the COVID-19 pandemic, n (%)
**Clinical anesthesia year 1**			
	0-5	2 (25)	5 (62.5)
	5-10	6 (75)	3 (37.5)
	>10	0 (0)	0 (0)
**Clinical anesthesia year 2**			
	0-5	3 (60)	3 (60)
	5-10	2 (40)	1 (20)
	>10	0 (0)	1 (20)
**Clinical anesthesia year 3**			
	0-5	4 (80)	3 (60)
	5-10	0 (0)	2 (40)
	>10	1 (20)	0 (0)

In total, 56% (10/18) of residents felt that attending virtual didactics was easy; 39% (7/18) of respondents felt that it was neither easy nor difficult. A majority of residents (72%, 13/18) reported that their engagement during virtual didactics was lower or much lower than that in in-person didactics. Besides question banks, textbooks, web-based videos, and formal didactics were the top 3 resources to enhance residents’ clinical performance and help their preparation for standardized assessments (eg, board exams).

**Figure 1:**
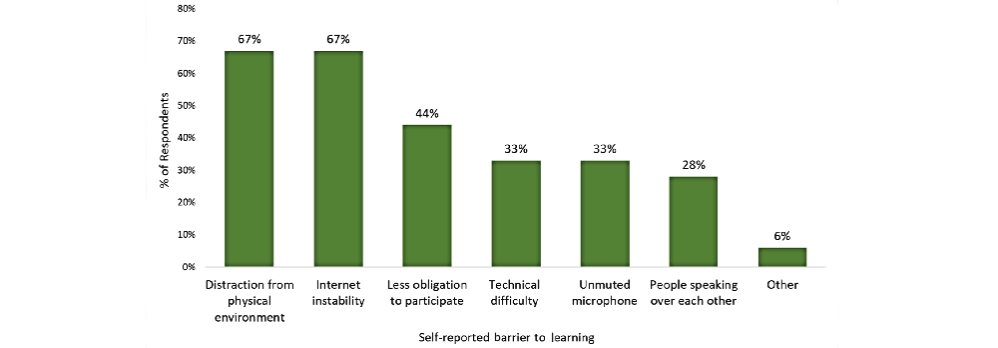
Self-reported barriers to web-based learning using virtual didactics.

As illustrated in Figure 1, barriers to engagement included distraction from the physical environment (67%, 12/18), internet instability (67%, 12/18), less obligation to participate (44%, 8/18), technical difficulty and unmuted microphones (33%, 6/18, each), and people speaking over each other (28%, 5/18). In addition, 72% (13/18) of residents felt that the pandemic negatively affected their interaction with their colleagues. Despite these limitations, most residents stated that they would like to keep a combination of virtual didactics including live Zoom lectures (56%, 10/18), prerecorded web-based didactics (56%, 10/18), and virtual ground rounds via Zoom (50%, 9/18) as the “new normal.”

## Discussion

### Principal Findings

The results of this pilot study demonstrate some of the negative impacts of the COVID-19 pandemic on medical education and residency training. Not only did residents report a reduction in hands-on patient care and bedside training in the operating rooms during the pandemic, but they also reported decreased interaction with peer residents, faculty, and other health care providers. Remarkably, the time spent on didactic activities did not change during the surge of the pandemic, and our residents were generally satisfied with their web-based learning opportunities, despite the many limitations associated with virtual lectures, conferences, and grand rounds. This reported comfort and satisfaction with web-based education is consistent with findings from other studies on the effects of the COVID-19 pandemic on medical education [11] and illustrates the ability of both residents and educators to effectively adapt to the significant changes that were brought forth by the COVID-19 pandemic. Although not specifically explored in this survey, these educational innovations included reformatting lectures and conferences to better fit the new web-based forum; recruitment of local, regional, and national speakers; and a focus on active leaning techniques, small group case discussions, and visual diagnostic and panel discussions. Specifically with respect to the field of anesthesiology, virtual didactics may have additional benefits to postcall residents who may not have previously been able to attend didactics in person but can attend virtually. This also applies to intensive care unit residents as well as those on external rotations.

Remote learning can present unique challenges for anesthesia trainees and residents. The videoconferencing software used in our institution (Zoom) allows annotation on a shared screen without interrupting the speaker. However, unintended interruptions from unmuted microphones and other technical difficulties such as internet instability can negatively impact the ability to remain attentive during virtual didactics, as was reported by our residents. Our residents were also distracted owing to physical environments that are inherent to web-based lectures and conferences. Most technical limitations can be addressed by simple modifications or upgrades of the conferencing software. For instance, distractions from unmuted microphones can be eliminated by activating the push-to-talk feature, which requires attendees to hold down a key to be unmuted. Speaking over each other can also be reduced if the speaker controls all microphones and determines when to open the session for questions or discussions. In contrast, environmental and pedagogic challenges may require a more sophisticated and innovative approach [12]. Examples include real-time facilitation by messaging discussion forums, question-and-answer polling, social feeds, and private notes to improve the experience and increase audience engagement. Large-group didactics can be replaced with the more interactive small-group case discussions and question-and-answer platforms.

Despite these software, internet, and environmental improvements, nevertheless, it is difficult to expect the same level of engagement with virtual learning as in-person didactics. Therefore, we expected that a majority of residents would prefer to eliminate virtual didactics after the pandemic and revert to traditional, face-to-face learning. To our surprise, however, a majority of our residents reported that they would like to keep virtual didactics, grand rounds, and web-based video learning as the new standard.

### Limitations

While the results of our pilot study provide new insights into the challenges and barriers associated with remote learning specific to anesthesia residents, our study has several limitations. Importantly, use of the survey methodology is associated with inherent limitations such as interpretation bias, social desirability bias, and lack of personalization. Moreover, our small sample is only representative of one academic institution during a surge in COVID-19 cases. The purpose of this study was to examine the effects of the pandemic on residency training experience, and it does not have the power to examine every aspect of web-based education. We did observe an increasing number of regional and national speakers during the completion of the survey and new approaches to improve the technology, but as these innovations and changes were not specifically examined in our study, we are unable to carry out further evaluation. Future studies are needed to address the impact of these innovations on resident education.

### Conclusions

Virtual didactics will certainly not replace bedside training, simulation training, or technical skill teaching, including ultrasound training, line placement, regional anesthesia, and—for all surgical specialties—hands-on surgical skill training. For didactic activities, nevertheless, residency programs may plan and design a hybrid curriculum that includes both in-person and virtual components, even beyond the COVID-19 pandemic. Larger studies are warranted to outline the specific barriers and opportunities for remote learning in different medical specialties. Innovations aimed at improving virtual didactics are also needed, as are studies to evaluate their efficacy and educational values within each medical field.
